# Origin of Low CO_2_ Selectivity on Platinum in the Direct Ethanol Fuel Cell**

**DOI:** 10.1002/anie.201104990

**Published:** 2012-01-02

**Authors:** Richard Kavanagh, Xiao-Ming Cao, Wen-Feng Lin, Christopher Hardacre, P Hu

**Affiliations:** School of Chemistry and Chemical Engineering, The Queen's University of BelfastBelfast BT9 5AG (UK); * School of Chemistry and Chemical Engineering, The Queen's University of BelfastBelfast BT9 5AG (UK)

**Keywords:** density functional calculations, electrochemistry, fuel cells, heterogeneous catalysis, platinum

The direct ethanol fuel cell (DEFC) represents one of the most exciting future clean energy solutions in modern research, because ethanol can be sustainably produced from biomass, is relatively nontoxic and, most importantly, has a high energy density.^1^–^8^ The exceptional energy density is due to the transfer of 12 electrons from ethanol during complete electrochemical oxidation (as opposed to six electrons from methanol or two from hydrogen). The practicality of such a device is contingent on its ability to selectively catalyze the total oxidation of ethanol to CO_2_.^9^, ^10^ However, the CO_2_ selectivity in the current ethanol fuel cells is very low, and the main products are acetic acid (resulting in the transfer of only four electrons) and acetaldehyde (only two electrons) in most systems reported.^11^–^13^ Herein, we address the origin of low CO_2_ selectivity in the DEFC, arguably the most important question to be answered in the field, using first-principles calculations.

The pioneering work on DEFCs can be traced back to the 1950s,^14^ but it was not until later that the selectivity was comprehensively investigated with IR spectroscopy showing CO_2_ to be a minor product.^15^ Behm and co-workers^1^ performed a thorough investigation on the selectivity under a wide range of conditions and employing a wide range of morphologies, and they convincingly showed that platinum catalysts exhibit selectivity towards CO_2_ in the region of 0.5–7.5 %, which is far short of the selectivity needed for economic implementation of the technology. This problem has proven difficult to surmount empirically. Recent work has made significant progress in terms of activity and selectivity,^2^ but further improvements are required. Theoretical studies have made considerable advances^2^, ^16^–^18^ and identified the platinum monoatomic step as the most likely site for total ethanol oxidation and concluded that the close-packed surfaces are unsuitable.^17^

Despite the extensive experimental and theoretical work that has been carried out, the inhibiting factors in CO_2_ formation remain unclear. There are good reasons for this: 1) the catalytic reactions occur on solid surfaces in the presence of a solvent, resulting in a system that is complex to understand at the molecular level; and 2) electrocatalysts operate at an applied potential (i.e. bearing charge), leading to more complications. Hence, it is extremely difficult to characterize the molecular-level surface processes by using experimental techniques, and it is also a huge computational challenge to realistically model the system. Without a clear understanding of the issue, strategies to overcome the problem remain limited to trial and error.

Although the selectivity problem has been identified, a fundamental understanding of the low CO_2_ selectivity observed is still missing. In fact, the low selectivity of CO_2_ in ethanol fuel cells is in contrast to the general consensus in chemistry: CO_2_ is significantly more stable than the major products acetic acid and acetaldehyde. According to the Bronsted–Evans–Polanyi (BEP) relationship in catalysis,^19^–^22^ the kinetics of any catalytic reaction is to some extent controlled by the thermodynamics of the reaction. In other words, one would expect the thermodynamically favored reactions to be faster kinetically. It is clear that the underlying reason behind the low CO_2_ selectivity is not only a key technological issue in the field but also a fundamental scientific question. Solving this long-standing puzzle will undoubtedly shed light on the selectivity of other systems in chemistry. Herein, we present results from first-principles simulations, based on one of the most realistic models of the catalytic processes to date, on the platinum surface in the presence of surface defects and, in doing so, explain the poor catalyst selectivity at the atomistic level.

All calculations reported herein were carried out using the VASP package^23^–^25^ (see the Supporting Information for computational details).^26^ Specific attention should be paid to the method by which the aqueous medium was considered. Modeling was achieved using Nose thermostat molecular dynamics (MD) simulations (*T*=353 K, 0.5 fs/step, 6000 steps). For these calculations, the DFT-optimized surface species were fixed, while an initial ice-like water structure was allowed to relax. Subsequently, six configurations were randomly selected from the last 200 time steps for each species and optimized by DFT, with the lowest-energy configuration being reported. In each case, the six calculated total energies were consistent to within 0.05 eV.

Considering that CO can readily be converted to CO_2_ in the presence of water,^17^, ^27^ we only calculated the pathway from ethanol to CO, and hence low CO_2_ selectivity is addressed as low CO selectivity herein. Furthermore, because acetic acid is the main product and CO_2_ is the desired product, herein we focus on the understanding of the production of acetic acid and CO from ethanol to shed light on the low CO_2_ selectivity. We first calculated all the feasible pathways for the formation of acetic acid and CO (the reaction scheme is shown in Scheme 1), with the minimum energy pathways being reported in each case (Scheme 2). The thermodynamic data and barriers for the minimum energy pathways are listed in Table 1. The corresponding energy profiles are shown in Figure 1. From the table and figures, we can see the following features: Firstly, the formation of acetic acid and CO are thermodynamically viable, with a general trend of increasing stability along the reaction coordinate. Secondly, no large barrier exists in either the acid or CO formation pathways, thus indicating that both processes are kinetically feasible. The calculated energy profiles for CO and acetic acid production are found to be in good agreement with those reported.^17^ The energy profiles clearly suggest that, on a clean surface, CO_2_ formation is both thermodynamically and kinetically competitive with acetic acid formation.

**Figure 1:**
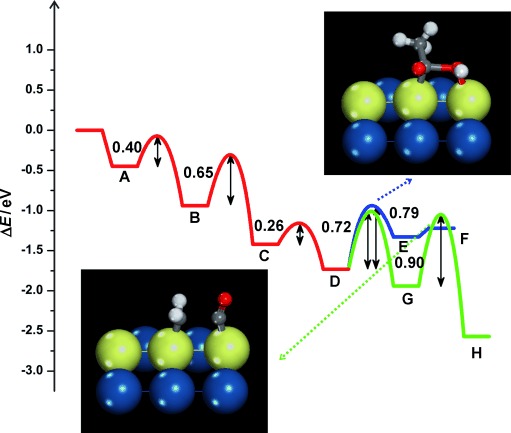
Energy profiles for the reactions shown in Scheme 2. Intermediate states (A–H) are defined in Scheme 2. The pathway highlighted in red (yielding adsorbed CH_3_CO (CH_3_CO_(ads)_), D) is common to both acetic acid and CO formation. The pathway highlighted in blue is associated with acetic acid production. The pathway highlighted in green is related to CO formation. The transition states of the key steps for acetic acid and CO formation are shown in the inserts. In the inserts, the Pt atoms are shown in dark blue except Pt atoms on step edge in yellow, C in gray, O in red, and H in white.

**Scheme 1:**
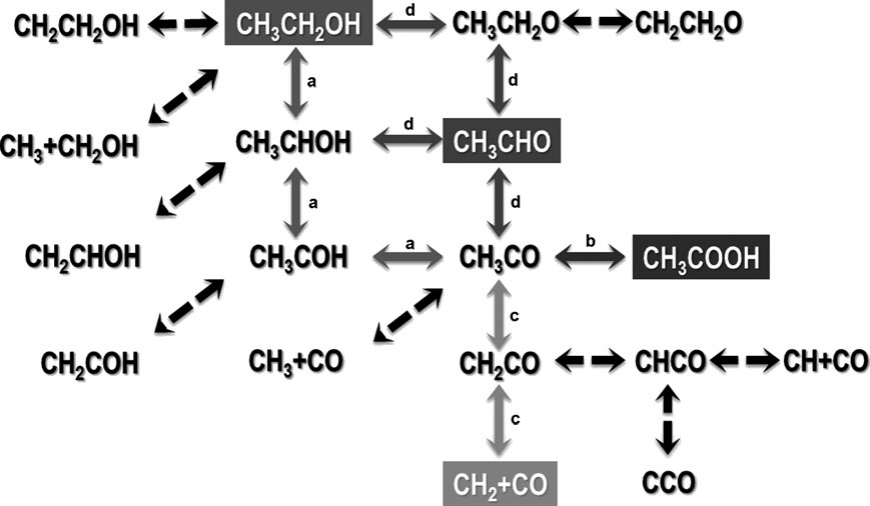
Reactions calculated for the formation of acetaldehyde, acetic acid and CO_2_. Since CO can readily be converted to CO_2_ in the presence of water (see text and Ref. 22, 27), the CO formation rather than CO_2_ was investigated herein. The full arrows indicate the common pathway (a), the acetic acid formation pathway (b), the CO formation pathway (c), and the acetaldehyde formation pathway (d). The dashed arrows indicate the unfavored reaction pathways.

**Scheme 2:**
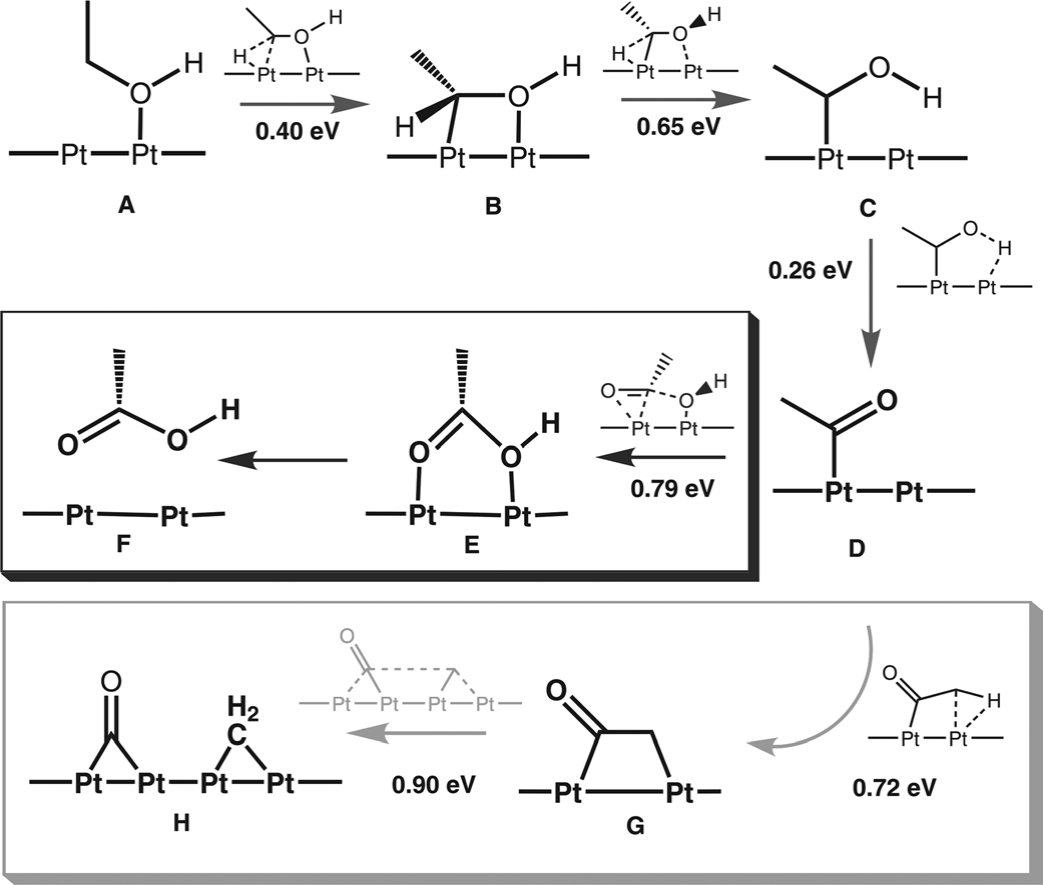
Surface reaction scheme for the located minimum energy pathways of acetic acid and CO formation. The transition state structures and associated energy barriers are illustrated for each step. The pathway via intermediate states A–D is common to both acetic acid and CO formation. The reactions in the upper box are related to acetic acid formation. CO formation reactions are shown in the lower box.

**Table 1:** Thermodynamic (Δ*E*) and kinetic data (*E*_a_) of the minimum energy pathways for acetic acid and CO formation.

	Δ*E* [eV]	*E*_a_ [eV]
CH_3_CH_2_OH_(g)_→CH_3_CH_2_OH_(ads)_	−0.45	–
CH_3_CH_2_OH_(ads)_→CH_3_CHOH_(ads)_	−0.94	0.40
CH_3_CHOH_(ads)_→CH_3_COH_(ads)_	−1.42	0.65
CH_3_COH_(ads)_→CH_3_CO_(ads)_	−1.73	0.26
CH_3_CO_(ads)_→CH_2_CO_(ads)_	−1.94	0.72
CH_2_CO_(ads)_→CH_2(ads)_+CO_(ads)_	−2.57	0.90
CH_3_CO_(ads)_+OH_(ads)_→CH_3_COOH_(ads)_	−1.33	0.79

However, these results appear to be at odds with existing experimental data regarding the selectivity towards CO_2_. To examine the system in more detail, we performed kinetic analyses (see the Supporting Information), from which the key steps in acetic acid formation and CO_2_ formation via CO oxidation were found to be the Reactions (1) and (2), respectively (highlighted in Figure 1).


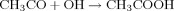
(1)


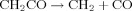
(2)

As such, the coupling of the acetyl and hydroxy species is the crucial step in acetic acid formation, whereas C–C bond cleavage is the key step to form CO and, thereafter, CO_2_. Importantly, there are no viable pathways for the further oxidation of acetic acid. In other words, our kinetic analyses show that the selectivity towards CO/CO_2_ formation versus that towards acetic acid formation in the system is determined by the competition between the elementary step of CH_2_CO→CO+CH_2_ and the step of CH_3_CO+OH→CH_3_COOH. With the key steps determined, we further investigated the effects of water and applied potential on acetic acid and CO_2_ formation to more effectively assess their selectivity in the real system. The effects of applied potential on the system have been studied in the last few years, and the principal effect has been found to be associated with the formation and speciation of the surface oxidants (OH and O) by the following reactions:^28^–^30^


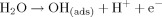
(3)


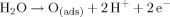
(4)

Importantly, not only do the relative and absolute concentrations of O and OH change with potential, but the presence of the oxidant has a significant effect on the reaction barriers. We calculated the effect of these surface oxidants on the C–C bond cleavage step by using oxidants at a coverage of 1/3 ML (ML=monolayer), which is in accordance with existing literature data.^29^ Note that in acetic acid formation, the presence of OH has already been explicitly taken into account [Equation (1)]. Also note that, while oxidant coverage will have an effect on all elementary steps, only the effects on the steps relevant to the selectivity have been calculated. The results summarized in Figure 2 show that the presence of surface oxidants will considerably increase the barrier of the C–C bond cleavage. From the barrier difference, it can be estimated that the rate of the crucial C–C bond cleavage step will be reduced by about two orders of magnitude in the presence of OH and about six orders of magnitude in the presence of O compared with the clean surface at a temperature of 333 K. Accordingly, these surface oxidants severely inhibit the catalysis to form CO and, thus, CO_2_. It is therefore the blocking effect, caused by these surface oxidant species, that is responsible for the low selectivity towards CO_2_ observed in real systems.

**Figure 2:**
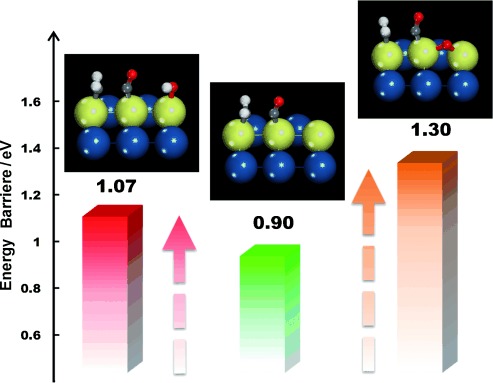
Effect of surface oxidants (O, OH) on the key step of CO formation, which is the C–C bond cleavage. The bars are the barriers of the key step of CO formation without oxidant (green), in the presence of OH (red), and in the presence of O (brown). The inserts above the bars show the corresponding transition states of the C–C bond cleavage. Pt atoms are shown in dark blue except Pt atoms on step edge in yellow, C in gray, O in red, and H in white. In the presence of OH or O, the barrier of C–C bond cleavage is considerably increased. From differences in barriers, the rate of CO formation can be estimated to be reduced by approximately six orders of magnitude in the presence of O compared to that in absence of oxidant.

It should also be noted that both barriers of the key steps for acetic acid (0.79 eV) and CO (1.07 eV) formation in the presence of OH are high for a system that is known to be active at room temperature. As such, and to gain more quantitative understanding of the system, we also considered the effect of the aqueous medium on the reaction kinetics. The energy barriers for the key steps [Equations (1) and (2)] in the presence of water medium were determined using MD calculations. The barrier associated with acid formation was reduced from 0.79 to 0.65 eV, while the barrier associated with C–C bond cleavage was decreased from 1.07 to 0.86 eV. It can be estimated from this data that, in the presence of the water medium, both the rates of acetic acid and CO formation are increased by approximately two to three orders of magnitude compared with those without water at a temperature of 333 K. This increase in rate is due to the relative stabilization of the high-energy transition states with respect to the intermediates through hydrogen bonding with the water molecules.^31^ The presence of water molecules causes considerable changes to the reaction kinetics; these changes bring the theoretical model into good agreement with experimental data and, perhaps more importantly, highlight the importance of considering the effect of the solvent medium when modeling liquid-phase catalytic systems.

It is clear from these results that, on a clean Pt surface with defects under low applied potentials, and thus low oxidant coverages, the formations of acetic acid and CO/CO_2_ are energetically favorable and, interestingly, comparable. This finding is consistent with the experimental data that shows that CO formation is indeed reasonably facile at low applied potentials, that is, for the clean surface with low concentrations of surface oxidant.^32^ An increase in potential causes an increase in oxidant surface coverage and, according to our results, will lead to a large reduction in the rate of C–C bond cleavage. However, to obtain turnover of the CO to CO_2_, surface oxidants are required. In the absence of surface oxidants, the strong adsorption of CO results in site blocking as observed on Pt catalysts used in direct methanol fuel cells.^33^, ^34^ These two competing processes explain the inability of pure platinum catalysts to act as efficient DEFC catalysts. Overall, the surface processes as a function of the applied potential can be summarized as follows:

1. At low potentials, CO formation occurs readily but, owing to the unavailability of oxidants, CO_2_ production is limited and CO effectively acts as a poisoning species.
2. At higher potentials, C–C bond cleavage is inhibited by the presence of oxidants, thus leading to reduced CO/CO_2_ production.

From these results, it is therefore unlikely that pure platinum-based DEFC catalysts will be sufficiently active for CO_2_ production to be practical. This study highlights the need for careful control of oxidant surface coverage that will allow facile C–C bond cleavage while still providing sufficient levels of CO oxidation. As demonstrated in recent experimental studies,^2^ this is likely to be most successful through the use of doping agents or, potentially, novel reaction media.

## Supplementary material

Detailed facts of importance to specialist readers are published as ”Supporting Information”. Such documents are peer-reviewed, but not copy-edited or typeset. They are made available as submitted by the authors.
