# Supplementary material for: Origin of Low CO2 Selectivity on Platinum in the Direct Ethanol Fuel Cell
Source: Angew Chem Int Ed Engl. 2012 Jan 2;51(7):1572–5. doi: 10.1002/anie.201104990 (PMC3625737; doi:10.1002/anie.201104990)
Supplement: Supplementary file 1 [file anie0051-1572-SD1.pdf]

Angewandte Chemie
Eine Zeitschrift der Gesellschaft Deutscher Chemiker

Supporting Information
© Wiley-VCH 2012
69451 Weinheim, Germany

Origin of Low CO_2 Selectivity on Platinum in the Direct Ethanol Fuel Cell**

Richard Kavanagh, Xiao-Ming Cao, Wen-Feng Lin, Christopher Hardacre, and P. Hu**

anie_201104990_sm_miscellaneous_information.pdf

Supporting Information

1. Computational Details

All calculations reported here were carried out using the VASP package with the use of a plane wave basis set.[1-3] Electron exchange and correlation terms are described with the use of the generalised gradient approximation (GGA) using the PBE functional.[4] Electron-ion interactions are described using projector-augmented wave (PAW) potential.[5] The convergence of the plane-wave expansion was obtained using a cut-off energy of 400 eV. The two dimensional Brillouin integrations were fulfilled using a (5 x 5 x 1) Monkhorst-Pack grid.[6] The ground state was obtained using Methfessel-Paxton smearing of 0.05 eV.[7] Kinetic data was obtained using a constrained minimization transition state search technique.[8-10]

The monoatomic step was modelled as a (3 x 1 x 1) (2 1 1) unit cell. A slab of four layers was employed, with the upper two layers being relaxed and the lower two in fixed geometry. Separation of slabs in the normal direction was achieved using a vacuum region of 12 Å. The aqueous medium was modelled using Nose thermostat molecular dynamics simulations (T = 353 K, 0.5 fs/step, 6000 steps). For these calculations, the DFT-optimised surface species were fixed, while an initial ice-like water structure was allowed to relax. Following MD calculations, 6 configurations were randomly selected from the last 200 time-steps for each species and optimised by DFT, with the lowest-energy configuration being reported. In each case, the 6 calculated total energies were consistent to within 0.05 eV, demonstrating that the systems had reached an equilibrium state.

2. Kinetics

The formation of CO_2 and acetic acid initially share the same minimum energy pathway until the formation of $CH_3CO_{(ads)}$. This indicates that the elementary steps from ethanol to $CH_3CO_{(ads)}$ are not crucial to the selectivity between CO_2 and acetic acid. Starting from $CH_3CO_{(ads)}$, two parallel pathways yield CO_2 and acetic acid, respectively. Given that CO can be readily converted to CO_2 in the presence of water[11], either the dehydrogenation of $CH_3CO_{(ads)}$ to yield $CH_2CO_{(ads)}$ or the C-C bonding cleavage, *i.e.* $CH_2CO_{(ads)} \rightarrow CH_{2(ads)} + CO_{(ads)}$ is likely to be the key step in CO_2 formation. Comparing these two elementary steps, the energy barrier associated with C-C bond cleavage (0.90 eV) is higher than that associated with the dehydrogenation of $CH_3CO_{(ads)}$ (0.72 eV). This suggests that the C-C bond cleavage is the key step of CO_2 formation. It is expected that the coupling of $CH_3CO_{(ads)}$ and $OH_{(ads)}$ be the key step for acetic formation as it is a unique surface reaction step. Hence, the competition between C-C bond cleavage and the coupling of $CH_3CO_{(ads)}$ and $OH_{(ads)}$ determines the selectivity between CO_2 and acetic acid.

3. Intermediate and Transition State Structures and Bond Lengths (Å)

$CH_3CH_2OH_{(ads)}$

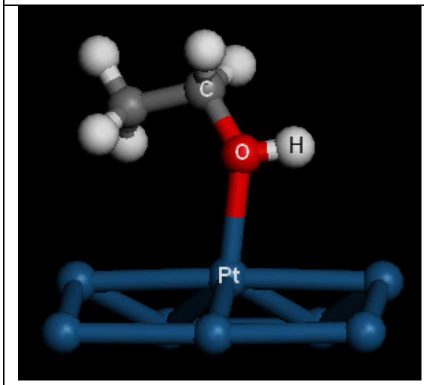

Pt – O	2.287
C – O	1.461
O – H	0.976

TS 1: $CH_3CH_2OH_{(ads)}$ → $CH_3CHOH_{(ads)}$

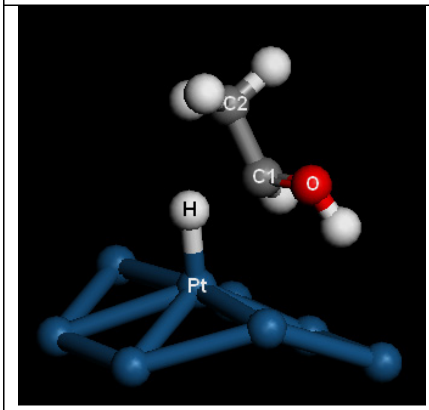

**C_1 – H**	**1.608**
Pt – H	1.617
Pt – C_1	2.461
C_1 – C_2	1.505
C_1 – O	1.358

$CH_3CHOH_{(ads)}$

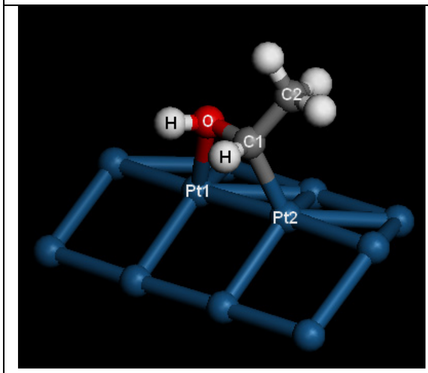

Pt_1 – O	2.292
Pt_2 – C_1	2.056
C_1 – C_2	1.510
C_1 – O	1.483
O – H_1	0.978
C_1 – H_2	1.103

TS 2: $CH_3CHOH_{(ads)}$ → $CH_3COH_{(ads)}$

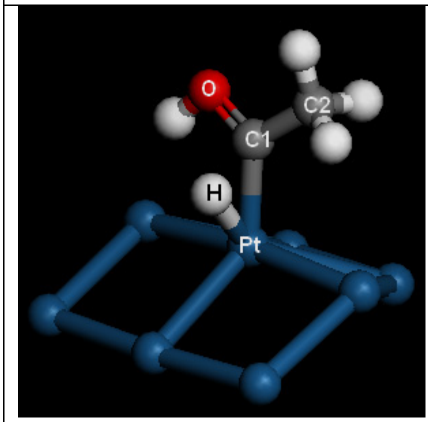

**C_1 – H**	**1.469**
Pt – H	1.698
Pt – C_1	1.973
C_1 – C_2	1.491
C_1 – O	1.327

$CH_3COH_{(ads)}$

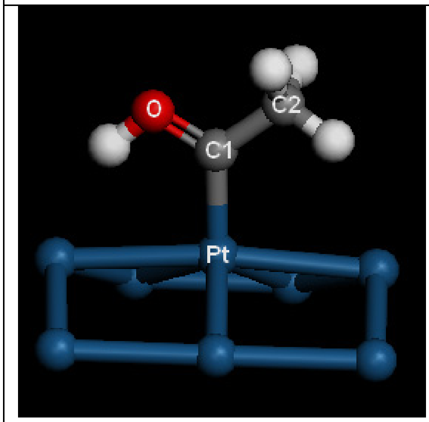

	Pt – C_1	1.926
	C_1 – C_2	1.494
	C_1 – O	1.319

TS 3: $CH_3COH_{(ads)}$ ➔ $CH_3CO_{(ads)}$

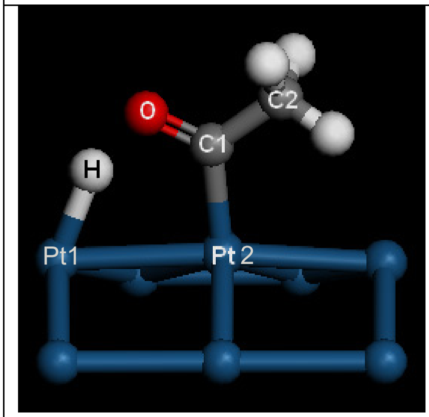

	**O – H**	**1.424**
	Pt_1 – H	1.682
	Pt_2 – C_1	1.974
	C_1 – C_2	1.510
	C_1 – O	1.255

$CH_3CO_{(ads)}$

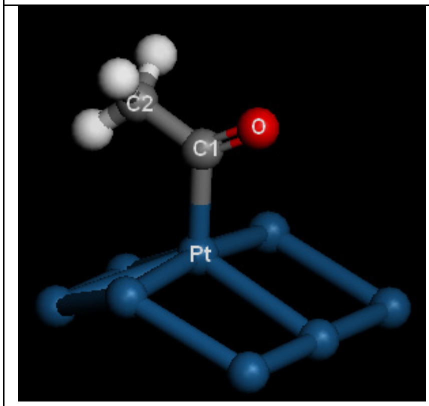

	Pt – C_1	2.001
	C_1 – C_2	1.514
	C_1 – O	1.211

TS 4: $CH_3CO_{(ads)}$ ➔ $CH_2CO_{(ads)}$

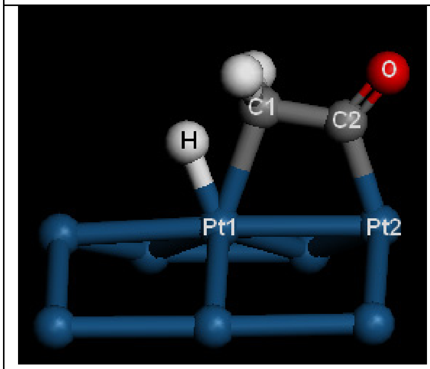

	**C_1 – H**	**1.441**
	Pt_1 – H	1.624
	Pt_1 – C_1	2.249
	Pt_2 – C_2	1.980
	C_1 – C_2	1.533
	C_2 – O	1.214

CH_2CO (ads)

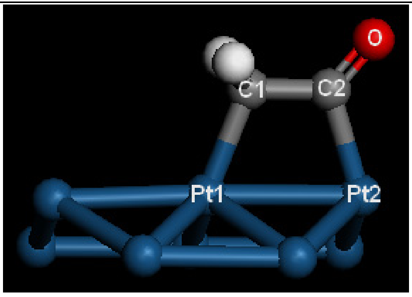

$Pt_1 - C_1$	2.070
$Pt_2 - C_2$	2.005
$C_1 - C_2$	1.499
$C_2 - O$	1.207

TS 5: CH_2CO (ads) ➔ CH_2 (ads) + CO (ads)

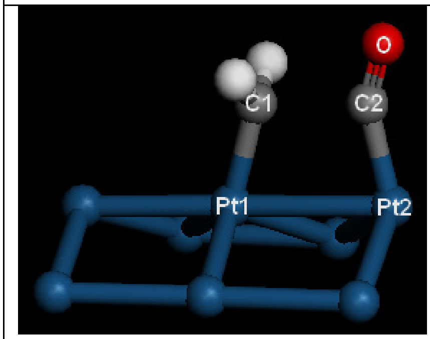

**$C_1 - C_2$**	**2.102**
$Pt_1 - C_1$	1.928
$Pt_2 - C_2$	1.911
$C_2 - O$	1.176

CH_2 (ads) + CO (ads)

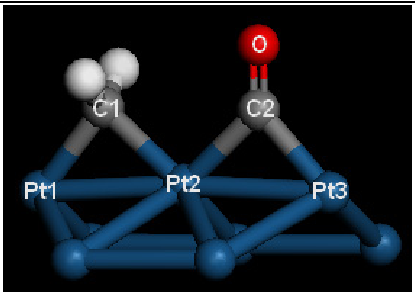

$Pt_1 - C_1$	2.012
$Pt_2 - C_1$	2.078
$Pt_2 - C_2$	2.063
$Pt_3 - C_2$	2.000
$C_2 - O$	1.184

TS 6: CH_3CO (ads) + OH (ads) ➔ CH_3COOH (ads)

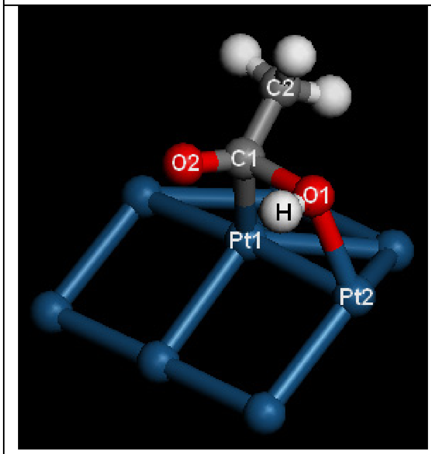

**$C_1 - O_1$**	**1.787**
$Pt_1 - C_1$	2.132
$Pt_2 - O_1$	2.096
$C_1 - C_2$	1.514
$C_1 - O_2$	1.227
$O_1 - H$	0.983

$CH_3COOH_{(ads)}$

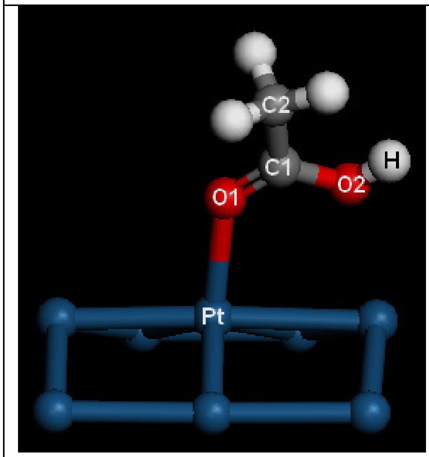

$Pt_1 - O_1$	2.284
$C_1 - C_2$	1.500
$C_1 - O_1$	1.231
$C_1 - O_2$	1.354
$O_2 - H$	0.978

TS 7: $CH_3COOH_{(ads)} \rightarrow CH_3COO_{(ads)}$

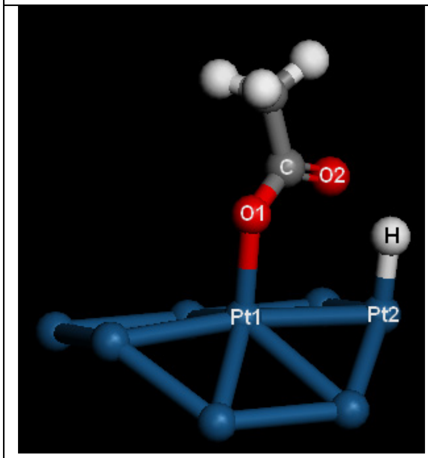

**$O_2 - H$**	**1.704**
$Pt_1 - O_1$	2.085
$Pt_2 - H$	1.581
$C - O_1$	1.303
$C - O_2$	1.256

$CH_3COO_{(ads)}$

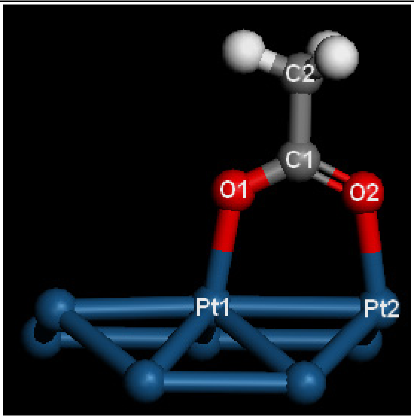

$Pt_1 - O_1$	2.086
$Pt_2 - O_2$	2.086
$C_1 - O_1$	1.279
$C_1 - O_2$	1.279
$C_1 - C_2$	1.506

(1) Kresse, G.; Hafner, J. *Phys. Rev. B.* 1993, **47**, 558.

(2) Kresse, G.; Hafner, J. *Phys. Rev. B.* 1993, **48**, 13115.

(3) Kresse, G.; Hafner, J. *Phys. Rev. B.* 1993, **49**, 14251.

(4) Perdew, J. P.; Burke, K.; Ernzerhof, M. *Phys. Rev. Lett.* 1996, **77**, 3865.

(5) Blochl, P. *Phys. Rev. B.* 1994, **50**, 17953.

(6) Monkhorst, H. J.; Pack, J. D. *Phys. Rev. B.* 1976, **13**, 5188.

(7) Methfessel, M.; Paxton, A. T. *Phys. Rev. B.* 1989, **40**, 3616.

(8) Alavia, A; Hu, P.; Deutsch, T.; Sylvestrelli, P. L.; Hutter, J. *Phys. Rev. Lett.* 1998, **80**, 3650.

(9) Michaelides, A.; Hu, P. *J. Am. Chem. Soc.* 2000, **122**, 9866.

(10) Liu, Z.-P.; Hu, P. *J. Am. Chem. Soc.* 2003, **125**, 1958.

(11) Reference (16) in main body.
